# Immunity and inflammation in cardiovascular disorders

**DOI:** 10.1186/s12872-023-03185-z

**Published:** 2023-03-23

**Authors:** Vennela Boyalla, Enrique Gallego-Colon, Michael Spartalis

**Affiliations:** 1 University of Manchester, Manchester, UK; 2 Escuela Naval Militar (E.N.M.), Pontevedra, Spain; 3 National and Kapodistrian University of Athens, 32 Tsakalof Ave, Athens, 10673 Greece

**Keywords:** Immunity, Inflammation, Cardiovascular, Pathophysiology, Molecular

## Abstract

Recent studies have developed our understanding of the role of the immune system and inflammation in Cardiovascular Disease (CVD), opening new avenues for risk stratification and therapeutic intervention. However, gaps in our knowledge remain. To address this issue, BMC Cardiovascular Disorders has launched a Collection on “Immunity and Inflammation in Cardiovascular Disorders”.

Cardiovascular diseases (CVDs) are the leading cause of death globally and pose an economic burden worldwide. Mounting evidence indicates that immune dysregulation and inflammation underlie many CVDs, including atherosclerosis, myocardial infarction, arrhythmias, pericardial disease, valvular heart diseases, cardiomyopathies, and heart failure. Promisingly, recent studies suggest that modulating inflammation may reduce cardiovascular (CV) events. However, the role of the immune system and inflammation in CVD is yet to be fully understood. To address this knowledge gap, *BMC Cardiovascular Disorders* welcomes submissions on this topic via the launch of a new Collection on **“**Immunity and Inflammation in Cardiovascular Disorders’’.

The host’s first line of defence against injury and infection is the innate immune system. It has been implicated in many CVDs, especially atherosclerosis. The immune-mediated cellular responses are modulated by controlling the timing and pace at which immune cells are recruited to the injured site. Neutrophils are the first immune cells recruited to the damaged tissue and represent the primary mechanism for local amplification of the inflammatory process [1]. Following the initial burst of neutrophils, other immune cells (monocytes, macrophages, dendritic cells, lymphocytes, mast cells) are also recruited to the damaged tissue. Monocytes and macrophages are the major cell types dominating the inflammatory process [2]. In an acute injury, as observed in myocardial infarction, the temporal dynamics of monocytes are controlled by cytokines and chemokines produced during this process, which in turn give rise to inflammatory and reparative macrophages [3]. Inflammatory macrophages amplify cytokine signals [IL-1β, IL-6, IL-12, nitric oxide] and release chemokines to recruit more inflammatory cells. In addition, they are involved with phagocytosis of cellular debris. Following, the clean-up phase, reparative macrophages promote deposition of new extracellular matrix, cell growth and regeneration. Macrophages also display anti-inflammatory profile, by serving as mediators in the resolution of the inflammatory process, as noted by the high production of IL-10, TGFβ, and IGF-1 [4], which assists in the progression from the inflammatory to the regenerative phase of repair. Inflammatory macrophages must be tightly regulated since the inflammatory cytokines and ROS species released can directly cause myofiber lysis, exacerbating the injury. The pivotal role of innate immunity, especially macrophages, is shown in a recent study showing that a clonal population of granulocytes carrying driver mutation related to haematopoiesis can cause tissue inflammation leading to atherosclerotic diseases [5].

The role of the adaptive immune system has shown an association with CVD risk due to a sustained and chronic inflammatory state. T and B cells in the atherosclerotic plaque have been hypothesized to generate an autoimmune-mediated process leading to vascular inflammation [6]. Interestingly, single-cell RNA, immunohistochemistry, and mass cytometry studies have shown that 25–28% of all leukocytes in atherosclerotic plaque are T-helper cells. CD3^+^ T-cells recruited mainly via CCR5 and CXCR6 contribute to fibrous cap stability. At the same time, CD4^+^ T cells can promote activation or dampening of T-cells and tissue-resident cells, provide B-cell help to induce antibody production, or exhibit cytolytic activity, thus modulating the inflammatory milieu at the plaque. Another interesting aspect is that inflammation and CVD risk are not limited to disease processes. Chronic inflammatory processes, as seen in periodontitis [7], irritable bowel syndrome [8], and rheumatoid arthritis [9] can potentially increase the risk of CVDs. Recent evidence suggests that arrhythmogenic diseases including atrial fibrillation, arrhythmogenic cardiomyopathy, cardiac sarcoidosis, and QT prolongation, can have an inflammatory component. Autoimmune and inflammatory-mediated cytokines, including IL-1, IL-6, and TNFα modulate the expression of ion channels by acting on cardiomyocytes resulting in a decrease of Potassium (K^+^) currents (I_Kr_, I_to_, and I_Ks_) and/or an increase of calcium current (I_CaL_) [10].

The role of innate and adaptive cells and associated molecular pathways involved in the development and outcome of CVD opens new unconventional treatment options. Consequently, considering the inflammatory involvement in CVDs, many clinical approaches have targeted both aspects of the innate and adaptive immune systems with inflammation-inhibiting agents. Several clinical studies, including the CANTOS, CIRT and COLCOT trials, provided evidence that modulation of the inflammatory response can potentially reduce the risk for major CV events. The CANTOS (Canakinumab Anti-inflammatory Thrombosis Outcomes Study) study suggests that reducing inflammation via antibody IL-1β targeting, without lowering the lipid levels can significantly reduce cardiovascular event rates [11]. In contrast, low-dose methotrexate did not show a reduction in plasma markers of inflammation in the Cardiovascular Inflammation Reduction Trial (CIRT). Nevertheless, new evidence supports the role of inflammation in atherosclerosis development as seen in the Colchicine Cardiovascular Outcomes Trial (COLCOT) [12]. The study showed that colchicine could significantly reduce the CV events in patients after myocardial infarction [13]. The underlying mechanism behind the reduction of CV events is likely to include direct inhibition of the NLRP3 inflammasome, TNF-α, IL-18, and IL-6, in addition to IL-1β [14]. Based on the clinical and experimental data, future therapies might be directed toward a combination of lipid-lowering and inflammation-inhibiting agents in patients with CVDs [15]. However, the risks and implications of inhibiting inflammation in the treatment of CVD in the host response to infection is yet another aspect to be studied.

Understanding cell-mediated immunology and molecular pathways behind CVDs is paramount in the development and implementation of effective anti-inflammatory and immune-modulating therapies for patients with CVD. Currently, we have state-of-the-art techniques at our disposal, including advanced in vivo imaging, genome-wide association studies, transgenic lineage tracing mice, mendelian randomization studies, and clinical trials to acquire a deep understanding of the immune landscape involving CVDs. In summary, we expect this article collection to gather evidences in both basic and clinical research in the CVD field to reinforce existing knowledge and open new therapeutic opportunities for treating these disorders.

## Data Availability

Not applicable.

## List of abbreviations

CANTOS: Canakinumab Anti-inflammatory Thrombosis Outcomes Study
CIRT: Cardiovascular Inflammation Reduction Trial
COLCOT: Colchicine Cardiovascular Outcomes Trial
CVD: Cardiovascular Disease
CV: Cardiovascular

## References

[1] Frangogiannis NG, Smith CW, Entman ML. The inflammatory response in myocardial infarction. Cardiovasc Res. 2002 Jan;53(1):31–47. 10.1016/s0008-6363(01)00434-5. 10.1016/s0008-6363(01)00434-5 11744011

[2] Arnold L, Henry A, Poron F, Baba-Amer Y, van Rooijen N, Plonquet A, Gherardi RK, Chazaud B. Inflammatory monocytes recruited after skeletal muscle injury switch into antiinflammatory macrophages to support myogenesis. J Exp Med. 2007 May 14;204(5):1057-69. doi: 10.1084/jem.20070075. 10.1084/jem.20070075 PMC2118577 17485518

[3] Tonkin J, Temmerman L, Sampson RD, Gallego-Colon E, Barberi L, Bilbao D, Schneider MD, Musarò A, Rosenthal N. Monocyte/Macrophage-derived IGF-1 orchestrates murine skeletal muscle regeneration and modulates Autocrine polarization. Mol Ther. 2015 Jul;23(7):1189–200. 10.1038/mt.2015.66. 10.1038/mt.2015.66 PMC4817788 25896247

[4] Frangogiannis NG. The inflammatory response in myocardial injury, repair, and remodelling. Nat Rev Cardiol. 2014 May;11(5):255–65. 10.1038/nrcardio.2014.28. 10.1038/nrcardio.2014.28 PMC4407144 24663091

[5] Jaiswal S, Ebert BL. Clonal hematopoiesis in human aging and disease. Science. 2019 Nov 1;366(6465):eaan4673. doi: 10.1126/science.aan4673. 10.1126/science.aan4673 PMC8050831 31672865

[6] Wolf D, Ley K. Immunity and Inflammation in Atherosclerosis.Circ Res. 2019 Jan18;124(2):315–327. doi: 10.1161/CIRCRESAHA.118.313591. 10.1161/CIRCRESAHA.118.313591 PMC6342482 30653442

[7] Humphrey LL, Fu R, Buckley DI, Freeman M, Helfand M. Periodontal disease and coronary heart disease incidence: a systematic review and meta-analysis. J Gen Intern Med. 2008 Dec;23(12):2079–86. 10.1007/s11606-008-0787-6. 10.1007/s11606-008-0787-6 PMC2596495 18807098

[8] Bigeh A, Sanchez A, Maestas C, Gulati M. Inflammatory bowel disease and the risk for cardiovascular disease: does all inflammation lead to heart disease? Trends Cardiovasc Med. 2020 Nov;30(8):463–9. 10.1016/j.tcm.2019.10.001. 10.1016/j.tcm.2019.10.001 31653485

[9] Skeoch S, Bruce IN. Atherosclerosis in rheumatoid arthritis: is it all about inflammation? Nat Rev Rheumatol. 2015 Jul;11(7):390–400. 10.1038/nrrheum.2015.40. 10.1038/nrrheum.2015.40 25825281

[10] Lazzerini PE, Capecchi PL, El-Sherif N, Laghi-Pasini F, Boutjdir M. Emerging Arrhythmic Risk of Autoimmune and Inflammatory Cardiac Channelopathies.J Am Heart Assoc. 2018 Nov20;7(22):e010595. doi: 10.1161/JAHA.118.010595. 10.1161/JAHA.118.010595 PMC6404431 30571503

[11] Ridker PM, Everett BM, Thuren T, MacFadyen JG, Chang WH, Ballantyne C (2017). Antiinflammatory therapy with Canakinumab for atherosclerotic disease. N Engl J Med.

[12] Tardif J-C, Kouz S, Waters DD, Bertrand OF, Diaz R, Maggioni AP (2019). Efficacy and safety of low-dose colchicine after myocardial infarction. N Engl J Med.

[13] Ridker PM, Everett BM, Pradhan A, MacFadyen JG, Solomon DH, Zaharris E (2019). Low-dose methotrexate for the Prevention of atherosclerotic events. N Engl J Med.

[14] Ruscica M, Corsini A, Ferri N, Banach M, Sirtori CR. Clinical approach to the inflammatory etiology of cardiovascular diseases.Pharmacol Res. 2020Sep;159:104916. doi: 10.1016/j.phrs.2020.104916. 10.1016/j.phrs.2020.104916 PMC7238995 32445957

[15] Gallego-Colon E, Wojakowski W, Francuz T. Incretin drugs as modulators of atherosclerosis.Atherosclerosis. 2018 Nov;278:29–38. doi: 10.1016/j.atherosclerosis.2018.09.011. 10.1016/j.atherosclerosis.2018.09.011 30248550

